# To study the utility of HER2 and Ki-67 as immunohistochemical prognostic markers in comparison to histopathological parameters and tumour, node and metastasis staging in colorectal carcinoma

**DOI:** 10.11604/pamj.2024.48.39.38445

**Published:** 2024-05-31

**Authors:** Suhit Naseri, Samarth Shukla, Sunita Vagha

**Affiliations:** 1 Jawaharlal Nehru Medical College, Datta Meghe Institue of Higher Education and Research, Sawangi, Wardha

**Keywords:** Colorectal carcinoma, immunihistochemistry, TNM staging

## Abstract

Colorectal Carcinoma (CRC) ranks among the most prevalent cancers globally, with significant variability in incidence rates across different regions. A shift towards a Westernized diet has been implicated in rising cancer rates, particularly in emerging nations. By 2020, CRC is projected to represent a notable proportion of global cancer cases and deaths. In India, CRC primarily affects individuals aged 45 to 84, with a higher incidence in males, commonly occurring in the rectum and sigmoid colon. Risk factors such as obesity, dietary factors, sedentary lifestyle, smoking, and alcohol use contribute to CRC development, especially in aging populations. Diagnosis involves various imaging modalities and histological assessments using Tumour, node and metastasis (TNM) and American Joint Committee on Cancer classifications. Recent advancements in targeted therapies like monoclonal antibodies against HER2 have shown promise in treating metastatic CRC. Immunohistochemistry markers like Ki-67 and HER2 play crucial roles in prognostic assessment and treatment planning. This study aims to investigate Ki-67 and HER2 expression in CRC, correlating with histological characteristics and prognostic factors.

## Introduction

Colorectal carcinoma (CRC) is one of the most prevalent malignancies globally, and it is the fourth leading cause of cancer-related death. CRC incidence rates vary greatly, with roughly 60% of cases identified in wealthy nations. The switch to a more Western diet has been linked to an increase in cancer rates in emerging nations [1]. In 2020, CRC will account for 10% of global cancer incidence and 9.4% of cancer-related deaths, trailing only lung cancer, which will account for 18% of fatalities. According to projections of population growth, ageing, and human progress, the global number of newly diagnosed CRC cases is predicted to reach 3.2 million by 2040.Based on projections of ageing, population expansion, and human progress, the worldwide number of new CRC cases is expected to reach 3.2 million by 2040 [2]. The majority of Indian research show that these tumours occur in people aged 45 to 84, with a male preponderance. The most likely locations for carcinomas are the rectum and sigmoid colon. Carcinoma is a malignant neoplasm of epithelial origin or a cancer originating from internal and external lining of the body. All cancers of the large intestine (colorectal cancer) originate from the cecum to the anus. Colon cancer, which extends from the cecum to the sigmoid (about 15 cm above the anal margin), and rectal cancer, which extends from the recto-sigmoid to the anus, are the two types of colorectal cancer. CRC originates as a benign polyp on the inner lining of the colon or rectum [3]. Unfavorable stressors like obesity, calorie rich diet, and diet devoid of fibre, minimal physical activity, cigarette smoking, and alcohol consumption, in addition to an ageing population in high-income nations, epithelial cells are exposed for a lengthened transit time. As a consequence, colorectal epithelium will be more susceptible to the impact of mutagenic compounds, thereby increasing cancer risk [4]. Patients may have a variety of signs and symptoms, including occult overt rectal bleeding, bowel changes, anaemia, or abdominal discomfort. Individuals over the age of 45 should have a colonoscopy if they experience rectal bleeding. CT colonography or MRI abdomen and pelvis are complimentary imaging methods for the diagnosis of colorectal cancer. MRI is used to do locoregional staging in rectal cancer. PET-CT imaging is also being used. The Tumour, node and metastasis (TNM) and American Joint Committee on Cancer classifications are used to predict the prognosis of newly diagnosed colorectal cancer (CRC) (8^th^ edition). Tumor extent, lymph node status, tumour grade, and lymphatic and venous invasion evaluation remain the major morphological prognostic variables. Tumor budding and tumour border configuration are crucial additional histological markers, however they are not considered critical in prognosis [5]. The availability of monoclonal antibodies against the vascular endothelial - and epidermal growth factor receptor has recently boosted the therapeutic arsenal. In mCRC, preclinical and clinical trials of Anti-HER2 targeted therapy have shown promising results. Because of the high mortality rate in advanced metastatic cancer, it appears that improved detection procedures are necessary. The concept of antibodies attaching selectively to antigens in biological tissues to detect antigens in cells of a tissue segment is utilized in Immunohistochemistry (IHC). The antibody-antigen binding can be seen in a variety of ways. In histology, immunohistochemistry is used to identify the presence of a particular protein marker that can help with tumour categorization and diagnosis. ErbB-2 is a receptor tyrosine-protein kinase that is also known as HER2. The prognostic biomarker human epidermal growth factor receptor 2 (HER2) is utilised to detect tumoral tissues. The transmembrane receptor protein is present on all normal cells and is involved in a wide range of biological processes such as cell proliferation and apoptosis, differentiation, and cell migration [4]. HER2 protein overexpression or gene amplification has been associated with higher stage, positive lymph node status and tendency for poor overall survival. Ki-67 as a prognostic predictor for well-known cancers. It has been established that its expression is inextricably bound to cell growth. Ki-67 expression and proliferation activity appear to rise dramatically, and it is essential in the majority of cell cycle stages. Elevated expression of Ki-67 in CRC is related with a lower survival rate, carcinogenesis, and cancer cell metastasis, suggesting that Ki-67 might be used as a prognostic biomarker in CRC patients [4]. The current study proposes to investigate the status of Ki-67 and HER2 expression in colorectal carcinomas in connection to prognostic parameters such as histological type, grade, tumour size, and lymph node status. The objectives were a) to confirm already diagnosed colorectal carcinoma by histopathological examination; b) to determine histological grades of CRC based on histopathological prognostic markers;m c) to determine staging of colorectal carcinoma by TNM classification based on American Joint Committee of Cancer (AJCC); d) to asses HER2 and Ki-67 expression in tumor tissues of colon and rectum by immunohistochemistry; e) to compare Ki-67 and HER2 expression with histopathological prognostic markers and TNM staging.

## Methods

**Review Question**: how do HER2 and Ki-67 expression levels correlate with histopathological parameters in colorectal carcinoma? What is the comparative prognostic value of HER2 and Ki-67 immunohistochemical markers versus TNM staging in colorectal carcinoma? Can HER2 and Ki-67 immunohistochemical analysis provide additional prognostic information beyond traditional histopathological parameters in colorectal carcinoma? How do HER2 and Ki-67 expression patterns vary across different stages of colorectal carcinoma according to TNM staging? What are the implications of incorporating HER2 and Ki-67 immunohistochemistry into the prognostic assessment of colorectal carcinoma alongside TNM staging and histopathological features?

**Study Design**: the current study is an observational, cross-sectional, retrospective, and prospective study that will last two years (June 2022 to June 2024), in the Histopathology and Immunohistochemistry division of the Department of Pathology, Jawaharlal Nehru Medical College, Sawangi (Meghe), in coordination with the Department of General Surgery, Acharya Vinoba Bhave Rural Hospital, Sawangi (Meghe). Approval will be obtained from Institutional Ethics Committee and informed consent will be taken from the patients participating in this study.

**Inclusion Criteria**: a) already diagnosed as colorectal carcinoma on histopathology; b) all operated cases of colorectal carcinoma; c) primary cases of colorectal carcinoma without any history of previous treatment; d) all patients with colorectal *carcinoma arising de novo*.

**Exclusion Criteria**: 1) all benign lesions of colon and rectum; b) all the already treated cases of colorectal carcinoma; c) all patients with colorectal carcinoma where the cancer is arising as a result of recurrence; d) patients with no histological confirmation of colorectal carcinoma.

**Participant's intervention comparison and outcomes (PICO)**: the information for PICOs (participants, intervention, comparison, and outcomes) is provided below;

**Population**: patients diagnosed with colorectal carcinoma (CRC).

**Intervention**: immunohistochemical analysis of HER2 and Ki-67 expression levels [6,7].

**Comparison**: comparison with histopathological parameters and TNM staging.

**Outcomes**: evaluation of the utility of HER2 and Ki-67 as prognostic markers in CRC and comparison with traditional histopathological parameters and TNM staging in predicting disease prognosis.

**Information sources**: the search will use sensitive topic-based strategies designed for each database. The search will be carried out in the following databases: Pubmed, Embase, Cinahl, Research Gate, Ajol, Google Scholar, Web of Science, Scopus and Cohrane Library. Only observational studies will be included.

**Search strategy**: ((“Colorectal Neoplasms”[MeSH] OR “Colorectal Carcinoma”[Text Word]) AND ("HER2"[MeSH] OR “HER2 Positive”[Text Word]) AND ("Ki-67 Antigen"[MeSH] OR “Ki-67”[Text Word]) AND ("Immunohistochemistry"[MeSH] OR “IHC”[Text Word]) AND ("Prognostic Markers"[MeSH] OR “Prognostic Factors”[MeSH] OR “Prognosis”[MeSH]) AND ("Histopathological Parameters"[MeSH] OR “Histopathology”[MeSH]) AND ("TNM Staging"[MeSH] OR “TNM Classification”[MeSH])).

**Sample size**: sample size calculation for a study estimating a population prevalence was described by Daniel in 1999. The calculation is intended to determine an adequate sample size to estimate the population prevalence with a good precision. The sample size calculation will be performed according to the formula suggested by Daniel.


$$\frac{{\left(Za/2\right)}^{2}*p*\left(1-p\right)}{d^{2}}$$

Where, “Z a/2” is the level of significance at 5% that is 95% confidence interval = 1.96 “p” is the prevalence of colorectal carcinoma = 0.2854 “d” is the desired error of margin = 7% = 0.07 “n” is the sample size. n = 1.96^2^x 0.2854 x (1-0.2854)/ 0.07^2^= 63.85 = Approximately 60-65 patients are needed in each study group. Study Reference: Consensus document for management of colorectal carcinoma by ICMR Formula Reference: (1999) Statistical formulas: Kappa statistics, Test statistics Software Used: SPSS 27.0 Version Level of study: Level III Sample Allocation: Random Selection of patients Study Design: Prospective Cross-sectional study.

**Figure 1:**
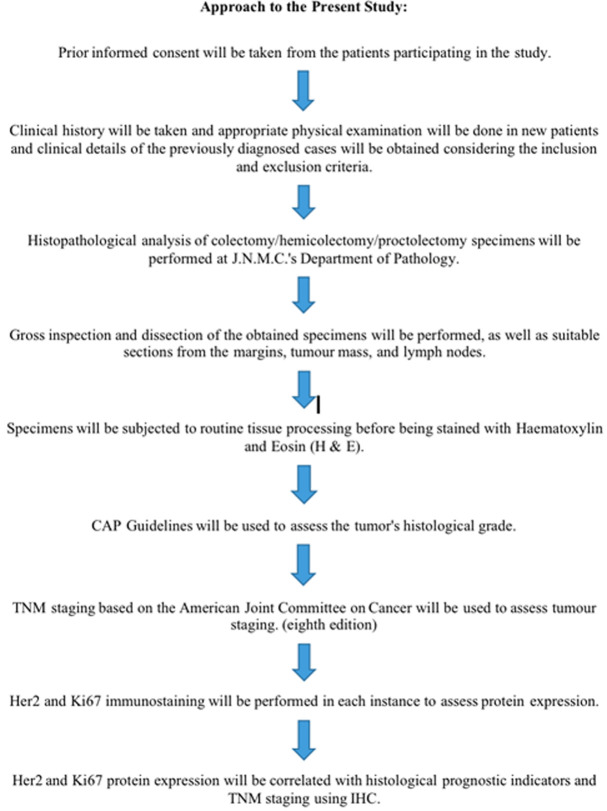
approach to the present study

**Approach to present study**: (Figure 1) the approach for the current study is detailed in the figure provided below. This figure outlines the step-by-step approach used in conducting the research and presenting the findings. It encompasses various stages of the study like determining the tumor staging using TNM (Table 1), Evaluation of expression of Ki-67 (Table 2) and HER2 (Table 3). This approach ensures transparency and clarity in how the study was conducted, facilitating reproducibility and reliability of the results.

**Table 1:** the stage of the tumor will be determined by TNM staging TNM Classification based on AJCC 8^th^ edition

Stage 0	Tis	N0	M0
**Stage I :**	T1 - T2	N0	M0
**Stage IIA :**	T3	N0	M0
**Stage IIB :**	T4a	N0	M0
**Stage IIC :**	T4b	N0	M0
**Stage IIIA :**	T1 - T2	N1/N1c	M0
	T1	N2a	M0
**Stage IIIB :**	T3 - T4a	N1/N1c	M0
	T2 - T3	N2a	M0
	T1 - T2	N2b	M0
**Stage IIIC :**	T4a	N2a	M0
	T3 - T4a	N2b	M0
	T4b	N1-N2	M0
**Stage IVA :**	Any T	Any N	M1a
**Stage IVB :**	Any T	Any N	M1b
**Stage IVC :**	Any T	Any N	M1c

**Table 2:** evaluation of expression of Ki-67 in colorectal carcinoma on Immunohistochemistry [4]

Immunoreactivity intensity scores	Immunoreactivity extent	Final score (immunostaning intensity x % of positive cells)
**Negative (0)**	<5% (0)	Negative Expression (-,0)
**Mild (1)**	6% to 25% (1)	Weak Positive Expression (+,1 - 3)
**Moderate (2)**	26% to 50% (2)	Moderate Positive Expression (+, 4 - 7)
**Strong/Intense (3)**	51% to 75% (3), 75% to 100% (4)	Strongly Positive Expression (+, 8 - 12)

**Table 3:** evaluation of expression of HER2 in Colorectal carcinoma on Immunohistochemistry [11]

Immunohistochemistry staining	Immunohistochemistry expected pattern	Immunohistochemistry classification
**No staining (0)**		Negative (-)
**Faint staining (1+) any cellularity**	Segmental or granular	Negative (-)
**Moderate (2+) in <50% of cells**	Any	Negative (-)
**Moderate (2+) in ≥50% of cells**	Circumferential, basolateral or lateral	Equivocal
**Intense (3+) in ≤10% cells**	Circumferential, basolateral or lateral	Negative (-)
**Intense (3+) in >10% <50% of cells**	Circumferential, basolateral or lateral	Positive (+)
**Intense (3+) in ≥50% of cells**	Circumferential, basolateral or lateral	Positive (+)

**Grossing techniques for colon and rectum specimen** [8]: surgery is the main stay of treatment of colorectal carcinoma. TNM staging of tumour is a major prognostic factor which helps decide further management. Derivation of TNM staging is entirely dependent upon a meticulous examination and appropriate sampling of surgical specimen by the pathologist (Table 1). 1) Unopened specimens in formalin were received along with proper clinical history. The specimens were checked for identification; 2) the nature of the surgical procedure was noted; 3) the length of the entire specimen was recorded; 4) palpation of the tumour was carried out on the outer aspect of the specimen; 5) the quality of total mesorectal excision before application of ink or opening the APR and AR specimens was assessed; 6) both aspects of specimen photographed for records purposes; 7) tumour site perforation looked for before inking; 8) the non-peritonealised surface was painted with ink with special reinforcement to the NPS related to the tumour. It is adviced to not paint the serosa; 9) upon being inked, the specimen should be opened from the anterior aspect starting from either ends of the tumor to 1 cm above and below the tumor; 10) the distances of both longitudinal resection margins from the tumour are noted; 11) the location of tumour was recorded with relation to the anterior peritoneal reflection in the rectosigmoid, AR and APR specimens; 12) the entire specimen was fixed in appropriate volume of formalin over the course of 48 hours; 13) upon adequate fixation, sample longitudinal mucosal resection margins; 14) document the size of tumour in two dimensions; 15) sample appropriate parts of the tumor as described above and submit for microscopy; 16) all lymph nodes were dissected and submitted; 17) examine the rest of the bowel segment for any abnormality; 17) mesorectum/peri-colonic fat was sampled; 18) sections to be taken; 19) four or five sections of the tumour, all inclusive of serosa and/or CRM; 20) all lymph nodes dissected off the specimen and submitted according to the level of the tumour; 21) longitudinal mucosal resection margins; 22) adjacent mucosa; 23) sample from any other grossly abnormal area.

**Materials**: a) the study will include approximately 60 resected specimens from confirmed and planned Colectomy specimen received in the Department of General Pathology, J.N.M.C; b) formalin fixed, paraffin embedded blocks of tumor masses from resected Colectomy specimens; c) 10% formalin; d) grossing instruments (grossing tray, knife, scalpel, measuring tape, plain forceps, toothed forceps); e) automated tissue processing assembly; f) haematoxylin & Eosin stain; g) HER2 and Ki-67 marker; h) glass slides (Blue Star®). Dimensions: 7.5x 2.5 centimeters; i) binocular research microscope.

**Staining Protocol: haematoxylin and eosin staining** [9]: a) colorectal carcinoma sections are deparaffinized in xylene: Three 10-minute shifts; b) sections dewaxing is performed. Sections are rehydrated using progressively higher grades of alcohol; c) bring sections to water; d) in a jar, stain for 10 minutes with Harris hematoxylin; e) wash for 2-3 minutes under running water; f) for a few seconds, separate in 1 percent acid alcohol (1 percent HCl in 70% alcohol); g) alkaline water was used for 5 minutes; h) for 1 minute, stain in 1% aqueous Eosin; g) dehydrate through 90% alcohol; h) fount in Dibutylphthalate Polystyrene Xylene (DPX).

**Procedure for immunohistochemistry as given by manufacturer (path in situ)**: a) 3 mm portions to be incubated for 1 hour at 60-70O C on charged slides; b) for 15 minutes each deparafinze using xylene for twice; c) hydrate through descending grades of alcohol as follows: 1) absolute alcohol- 2 changes, five minutes each; 2) 90% alcohol- 5 minutes; 3)70% alcohol-5 minutes; 4) wash in distilled water, two changes, 2 minutes each; 5) antigen retrieval for 15 -20 mins in MERS. pH of retrieval buffer (6,8 or 9.5) as per the marker; 6) wash in distilled water, two changes, 2 minutes each; 7) wash in PBS /TBS for 2 minutes; 8) endogenous peroxidation is inhibited by administering H2O2 to the area for 5 minutes. Wash in the wash buffer for 2 minutes twice; 9) for 30 minutes, place the HER2/Ki-67 primary antibody in a moist chamber. Then, wash twice in the wash buffer for 2 minutes each; 10) keep the Polyexcel Target binder reagent for 12 minutes. Wash in two different buffers for two minutes each; 11) incubate Polyexcel HRP for 12 minutes. Wash with buffer for 2 minutes, then change; 12) add functioning DAB chromogen (1ml DAB Buffer + 1 drop DAB Chromogen, mix thoroughly) and leave for 2-5 minutes before washing in distilled water; 13) hematoxylin counterstain for 30 seconds, then rinse with water; 14) dehydrate (70, 90, and absolute), clear (xylene), and mount as standard.

## Discussion

### Methodology of Interpretation

**Interpretation based on histologic grade** [10]: a variety of colorectal cancer grading systems have been proposed, however there is no single generally acknowledged and regularly utilised grading criteria. Most tumours are classified into three or four grades: a) Grade 1 (G1) - Well-differentiated; b) Grade 2 (G2) - Moderately differentiated; c) Grade 3 (G3) - Poorly differentiate; d) Grade 4 (G4) - Undifferentiated.

Despite high interobserver heterogeneity, multivariate analysis has repeatedly demonstrated that histologic grade is a stage-independent predictive predictor. High tumour grade, in particular, has been shown to be a poor predictive factor. The use of a two-tiered grading system for colorectal cancer is recommended in view of its established predictive efficacy, relative simplicity, and consistency. The following grading standards are proposed based only on gland development. A) low grade: >/= 50% gland formation; b) high grade: < 50% gland formation.

**Statistical analysis:** it will be carried out by ‘chi square test’ by analyzing the relationship of Ki-67 and HER2 protein expression in colorectal carcinoma. Multiple linear regression analysis is performed to determine the relative elements that contribute to metastasis. A value of P <0.05 considered to indicate statistical significance.

**Scope**: due to its high prevalence and mortality rate, colorectal cancer (CRC) has evolved into a global issue for public health. Immunohistochemical analysis and protein markers such as HER2 and Ki-67 are now being used to enhance the identification of individuals who are more likely to have a poor clinical outcome and hence benefit from early detection.

**Limitations**: 1) inter-observer and intra-observer variability; 2) technical errors while processing can influence the interpretation of immunostaining.

**Observation and results**: they will be collected and combined together over the period of two years and will be analyzed statistically.

**Trial registration number:** this study is registered with the Clinical Trials Registry - India (CTRI) and the Trial number is CTRI/2023/05/053165.

## Conclusion

The results of an IHC research employing HER2 and Ki-67 to identify HER2 and Ki-67 protein expression and correlate with histological prognostic indicators and TNM staging will be used to make conclusions.
